# Does Delayed Ligament Reconstruction Surgery Lead to Poor Outcomes in Patients With Multiligament Knee Injuries?

**DOI:** 10.7759/cureus.34219

**Published:** 2023-01-25

**Authors:** Aatif Mahmood, Hafiz M Umer, Arjun Paramasivan, Khurram Sheharyar, Rana Tahoun, Raghuram Thonse

**Affiliations:** 1 Orthopaedics and Trauma, Countess of Chester Hospital NHS Foundation Trust, Chester, GBR

**Keywords:** gender, obesity, knee, reconstruction, multiligament

## Abstract

Background and objective

Multiligament knee injuries (MLKIs) are difficult to treat, and poor outcomes have been reported after conservative management. Controversy exists as to how to surgically manage these complex injuries. The aim of our study was to evaluate the midterm patient-reported outcomes after delayed multiligament knee reconstruction.

Methods

This was a review of a prospectively kept database of all patients undergoing surgery for multiligament reconstruction at a single institution. A total of 51 patients were included in the study, with a median follow-up of 48 months. In addition to the patient-reported outcomes, we also looked at other factors that could potentially affect the outcomes.

Results

At the final follow-up, there was no significant difference between the range of motion (ROM) of the injured knee compared to that of the healthy knee. Patients having surgery >6 months after injury had similar outcomes when compared to those having surgery <6 months post-injury. Female patients and patients with a BMI >30 had inferior outcomes.

Conclusion

Good midterm results can be expected after delayed multiligament knee reconstruction. Patients with a BMI >30 and female patients were observed to have inferior outcomes.

## Introduction

Multiligament knee injuries (MLKIs) are uncommon and account for only 0.02-0.20% of all orthopedic injuries [1]. MLKI is associated with significant morbidity, and affected patients may continue to experience pain and instability several years after the original injury [2,3]. Concurrent tibial plateau fractures and neurovascular injuries can further complicate these injuries [2]. Various mechanisms have been described in terms of the cause of these injuries, and knee dislocation as a result of high-energy trauma such as road traffic accidents (RTAs) and sports injuries can lead to MLKI [4]. Low-energy injuries in morbidly obese patients may also lead to knee dislocations and MLKI [5].

Literature has reported poor outcomes with non-operative management of these injuries, leading to pain and residual instability [6-8]. Most surgeons, therefore, advocate operative treatment for these complex injuries. Operative treatment is associated with improved postoperative Lysholm scores, range of motion (ROM), and higher rates of return to work and pre-injury sports activities [9].

Due to the rarity of multiligament knee injuries, the treatment and rehabilitation of patients remain challenging. No protocols have been standardised so far for the treatment of such injuries due to the lack of larger randomised control trials. There is a paucity of evidence in the literature regarding the outcomes of these injuries. Also, controversy exists regarding several aspects of operative management: type of surgery (i.e., repair versus reconstruction); timing of surgery (i.e., early versus late), and single-stage versus staged surgery [2,10].

The primary aim of this study was to assess the midterm outcomes of single-stage multiligament reconstruction surgery to determine whether early or delayed treatment results in better outcomes. The secondary aim of this study was to examine the patient factors that may adversely affect the outcomes of surgery for MLKI.

## Materials and methods

This was a review of a prospectively collected database on all consecutive patients presenting with an MLKI, who underwent reconstruction surgeries at a district general hospital in the UK. All surgeries were performed by a single qualified sports knee surgeon (RT) between 2012 and 2018. The inclusion criteria were as follows: (a) injury to at least two stabilising knee ligaments, (b) single-stage reconstruction, (c) age >18 years, and (4) closed injury. The exclusion criteria were as follows: (a) previous history of knee ligament surgery, (b) associated fracture of the tibial plateau, (c) previous meniscus surgery, (d) patients who were lost to follow-up, (e) incomplete medical records, (f) bilateral knee injuries, and (g) avulsion injury or any injury requiring simple repair and not reconstruction, as this study purely intended to look at outcomes of reconstruction.

The data were collected from patients at the time of presentation and during at least two years of the follow-up period. Electronic medical records were reviewed to obtain demographic data and imaging reviews. Patients having surgery >6 months were delayed referrals to our service and were not deliberately delayed for surgery.

Diagnosis

Along with detailed history, physical examination, radiographs, and MRI data were used to establish the diagnosis of multiligament injuries. On-table stress radiographs were used to complete the evaluation of damaged ligaments.

The medial collateral ligament (MCL) was evaluated with a valgus stress test. A difference of <3.2 mm was taken as normal, while that of 3.2 to <9.8 mm was deemed a complete superficial MCL tear, and a difference of >9.8 mm was consistent with a complete tear of all medial-sided structures [10]. The fibular collateral ligament (FCL) was assessed with a varus stress test. A difference of <2.7 mm was regarded as normal, while that of 2.7 to <4 mm was considered an isolated FCL tear, and a difference of >4 mm was deemed a complete posterolateral corner (PLC) tear. MCL and FCL reconstruction was done on patients with ≥3.2-mm medial gaping on the valgus stress test or ≥2.7 mm of lateral gaping difference on varus stress testing respectively.

Preoperative physiotherapy

All patients underwent preoperative physiotherapy to improve their ROM and maintain muscle function till the time of surgery.

Surgery

Lateral Collateral Ligament (LCL)

A lateral longitudinal incision was made. The peroneal nerve was identified and protected. A fibular tunnel hamstring autograft was passed in a figure of 8/Larsen type repair. An image intensifier was used to establish the isometric point at the lateral epicondyle. In the initial six cases, for the first two years, a 40 mm x 6.5 screw with a spiked washer was used for fixing the other end of the graft. Later, 26 cases of fixation were achieved by a 6 or 7-mm Biosure PEEK screw (Smith & Nephew, London, UK).

Medial Collateral Ligament (MCL)

Two medial incisions at two attachments of MCL (distal oblique) were made. A neoligament (800 x 30 mm) was used. It was fixed to both MCL attachments after passing under the correct layer while retaining hamstrings (to avoid posteromedial corner laxity). Proximal fixation was achieved with a Biosure PEEK screw (Smith & Nephew) and distal with Titanium (Biomet) suture anchors (after preparing the bed to expose underlying cancellous bone). An additional stitch was placed at 1 cm distal to the joint line to mimic deep MCL function. All collateral ligaments underwent reconstruction instead of repair (the fully threaded cancellous fixation screw used was slightly different in the first few surgeries - interference screw after the first few years. Initially, a screw with a spiked washer was used for femoral side fixation for collateral ligament reconstruction).

Anterior Cruciate Ligament (ACL)

A longitudinal incision over the hamstring origin was done. Gracilis and semitendinosus tendons were harvested. Grafts were cleaned and whip-stitched. The entry point for the femoral tunnel was marked with a bone pick (10.30 for the right and 1.30 for the left). Beath pin and 4.5-mm drill were passed. Tunnel length was measured and an appropriate Endobutton (Smith & Nephew) was used. The tibial jig was positioned under vision. The graft was loaded onto the Endobutton and passed across the tibial and femoral tunnels. Endobutton was flipped and knee cycled to tension graft. Tibial fixation was achieved with an RCI screw (Smith & Nephew) inserted over the flexible guide wire. Additional femoral fixation with an RCI screw was employed in most cases to ensure adequate graft tension as well as to allow early mobilisation.

Posterior Cruciate Ligament (PCL)

Using a 70-degree knee arthroscope, the tibial and femoral tunnels were drilled using guides (Smith & Nephew Acutrack instruments). Gore Smoother was used to pass the graft (hamstring) through the tibial tunnel. The knee was held in 30 degrees of knee flexiongraft, which was then passed through femoral tunnels and fixed on both ends with RCI screws (Smith & Nephew).

All patients were assessed for the posteromedial corner (PMC) and PLC laxity and the need to reconstruct these. If necessary, PLC was reconstructed based on the methods described or Laprade’s method [11]. We did not have any patients who required PMC reconstruction. Nine patients required PLC reconstruction. They belonged to the ACL/LCL or ACL/LCL/PCL group and a separate analysis was not carried out on them.

Postoperative rehabilitation and physiotherapy

Physiotherapy was initiated on day one postoperatively with quadriceps muscle activation. All patients with MCL reconstruction were partial weight-bearing (PWB) and had 0-90 degrees of flexion in a brace for six weeks. Then, they had full ROM and full weight-bearing till three months when the brace was discarded. For those with lateral ligaments (LCL and PLC), toe-touch weight-bearing and 0-90 degrees of flexion in a brace for six weeks followed by further six weeks in a brace of full flexion and PWB were employed. Within three months postoperatively, crutches and braces were gradually discarded.

Postoperative review

The assessment was done by a surgeon or physiotherapist in the review clinic. This included evaluating the ROM compared to the opposite knee. Subjective assessments were done with Tegner Lysholm scores [12], International Knee Documentation Committee (IKDC) [13] scores, and Knee Injury and Osteoarthritis Outcome (KOOS) scores [14]. In all these scoring systems, a higher score represents a better function.

Statistical analysis

Data analysis was performed using IBM SPSS Statistics v 26.0 statistical analysis software (IBM Corp., Armonk, NY). Paired t-tests were used for groups before and after intervention whereas unpaired t-tests were used for independent groups. ANOVA tests were used to compare more than two groups. A p-value of 0.05 was considered statistically significant. Multiple regression was used to identify independent predictors of poor scores across the entire study population by adjusting for confounding variables.

## Results

A total of 59 consecutive patients underwent MLKI reconstruction between 2012 and 2018. Eight patients had incomplete data and hence were excluded from the study. The mean age of the patients at the time of surgery was 30.6 ± 10.7 years, with 37 (72.5%) males and 14 (27.5%) females. The mean time from injury to surgery was ­­­10 ± 8.9 months. The mean follow-up was 46 ± 9.12 months. Table 1 shows the demographic data of the study participants. Associated injuries included articular cartilage injuries (n=10, 20%), meniscus tears (n=14, 27%), and peroneal nerve injuries (n=1, 2%). Cartilage injuries were managed with chondroplasty (n=8, 80%) and microfracture (n=2, 20%). Treatments for meniscus tears were partial meniscectomy (n=8, 57%) and meniscus repair (n=6, 42%). The number of cartilage injuries and associated treatments were small and hence a separate analysis was not carried out. Overall, there was a significant improvement in postoperative Tegner Lysholm, IKDC, and KOOS scores (Table 2).

**Table 1:** Patient demographics SD: standard deviation; BMI: body mass index; ACL: anterior cruciate ligament; PCL: posterior cruciate ligament; MCL: medial collateral ligament; LCL: lateral collateral ligament

Variables	Values
Age, years, mean ± SD	30.6 ± 10.7
Age group, years, n (%)	
<30	25 (49.0%)
30-45	20 (39.2%)
>45	6 (11.8%)
Gender, n (%)	
Male	37 (72.5%)
Female	14 (27.5%)
Side, n (%)	
Right	34 (66.7%)
Left	17 (33.3%)
BMI, kg/m^2^, n (%)	
<30	33 (64.7%)
≥30	18 (35.3%)
Mechanism of injury, n (%)	
Fall	9 (17.6%)
Sports injury	23 (45.1%)
Road traffic accident	12 (23.5%)
Other	7 (13.7%)
Type of injury, n (%)	
ACL + MCL	20 (39.2%)
ACL + LCL	21 (41.2%)
ACL + MCL + LCL	6 (11.8%)
ACL + MCL + LCL + PCL	1 (2.0%)
Time from injury to surgery, months	
Mean ± SD	10 ± 8.9
≤6 months, n (%)	21 (41.2%)
>6 months, n (%)	30 (58.8%)
Follow-up duration, months	
Mean ± SD	46 ± 9.12
Median	48

**Table 2:** Overall results SD: standard deviation

Scoring system	Preoperative, mean ± SD	Postoperative, mean ± SD	Significance (p-value)
Tegner Lysholm Knee Scoring Scale	41.69 ± 17.8	81.5 ± 14.9	0.001
Knee Injury and Osteoarthritis Outcome Score	45.25 ± 17.3	81.06 ± 11.5	0.001
International Knee Documentation Committee subjective knee evaluation form	36.02 ± 15.8	78.18 ± 11.3	0.001

Surgical timing did not affect the outcomes. BMI >30 and female gender were noted to be significant contributors towards lower scores (Table 3). There was no significant difference in the preop scores between males and females (Table 4). Reconstruction for more than two ligament ruptures had significantly poorer scores when compared to two-ligament reconstructions. When comparing among two-ligament reconstructions, injuries involving LCL had significantly worse outcomes than MCL in terms of the Tegner Lysholm Knee Scoring Scale. All surgeries were single-staged. There were no major complications in terms of ligament failure. Two patients had superficial surgical site infections, which were treated with oral antibiotics with no further sequelae. One patient had preoperative peroneal nerve injury, which was managed conservatively and, at the latest follow-up, the recovery was almost complete. Two patients had prominent screws in the MCL reconstruction group, which were removed as day case surgeries. Table 3 further analyses the possible factors impacting the outcomes. All 51 patients returned to their previous employment, with 32 (62%) returning to high-impact sports, e.g., football.

**Table 3:** Factors impacting the outcomes BMI: body mass index; SD: standard deviation; IKDC: International Knee Documentation Committee; ACL: anterior cruciate ligament; PCL: posterior cruciate ligament; MCL: medial collateral ligament; LCL: lateral collateral ligament

Variables	Tegner Lysholm Knee Scoring Scale, mean ± SD	P-value	IKDC subjective knee evaluation form, mean ± SD	P-value	Knee Injury and Osteoarthritis Outcome Score (KOOS), mean ± SD	P-value
BMI, kg/m^2^ (N=51)						
>30 (n=18)	72.0 ± 16.9	0.003	72.5 ± 12.1	0.007	73.7 ± 11.9	0.002
≤30 (n=33)	86.7 ± 10.8		81.27 ± 9.6		85.1 ± 9.1	
Gender (N=51)						
Male (n=37)	84.7 ± 11.8	0.009	80.5 ± 10.6	0.014	83.1 ± 9.8	0.036
Female (n=14)	72.9 ± 18.9		72.0 ± 11.0		75.6 ± 13.9	
Type of repair (two versus more than two ligaments; N=51)						
2 (n=44)	84.39 ± 11.7	0	79.6 ± 10.2	0.025	82.8 ± 9.8	0.007
>2 (n=7)	63.14 ± 20.1		69.4 ± 14.5		70.4 ± 15.9	
Time to surgery, months (N=51)						
<6 (n=21)	77.8 ± 17.9	0.138	77.4 ± 14.0	0.696	78.5 ± 13.5	0.189
≥6 (n=30)	84.1 ± 12.0		78.8 ± 9.1		82.8 ± 9.6	
Medial versus lateral ligament repair (ACL/PCL & MCL vs. ACL/PCL & LCL) – comparison among reconstructions involving maximum 2 ligaments (N=44)						
ACL/PCL & LCL (n=25)	80.3 ± 13.5	0.028	76.7 ± 10.8	0.108	80.5 ± 10.2	0.21
ACL/PCL & MCL (n=19)	88.3 ± 8.2		81.7 ± 10.2		84.3 ± 9.1	

**Table 4:** Comparison of preoperative scores by gender

Scoring system	Male (n=37)	Female (n=14)	P-value
Tegner Lysholm Knee Scoring Scale	43.7	36.5	0.203
International Knee Documentation Committee subjective knee evaluation form	37.2	32.8	0.384
Knee Injury and Osteoarthritis Outcome Score	46.4	42.1	0.436

## Discussion

The optimum treatment strategy for MLKIs remains a subject of controversy. Our study has shown good midterm functional outcomes with a delayed reconstruction approach for the management of MLKIs. The median follow-up duration of our participants was 48 months. Female sex and higher BMI appeared to be associated with inferior outcomes.

While surgical treatment is associated with better outcomes, controversy exists regarding the timing of surgery. A review of the literature has suggested three different approaches: acute, staged, and delayed [1-2,15]. Benefits of early surgery include easy-to-identify anatomical structures without scarring and the ability to repair ruptured ligaments. However, this approach carries the risk of arthrofibrosis [1-2] and the need for further surgical intervention [16]. In addition, there is a risk of compartment syndrome due to fluid extravasation from the torn capsule [17]. Staged treatment involves treating the extra-articular structures acutely and a delayed reconstruction of intra-articular cruciate ligaments. This approach necessitates another anesthesia and prolonged rehabilitation [1-2]. Delayed reconstruction may enable us to avoid surgery on extra-articular structures that may heal with scarring and without surgery and is associated with a better ROM in the knee [1-2].

In their meta-analysis, Mook et al. [16] noted that patients undergoing acute surgery had more flexion deficits. However, a more recent meta-analysis involving 2,585 patients showed no difference in clinical and functional outcomes when comparing acute and delayed surgical intervention [18]. In our study, there were no cases of knee stiffness needing manipulation under anesthesia or arthroscopic adhesiolysis.

Obesity is considered a risk factor for multiligament injuries. Ridley et al. have shown that a high BMI not only makes individuals prone to MLKIs by low-energy mechanisms but also increases the risk of complications [19]. Another study by Lian et al. [20] reported that patients with higher BMI are at a significantly higher risk of complications and poorer outcomes. In our study, we noted that a BMI of >30 Kg/m^2^ was associated with significantly lower Tegner, IKDC, and KOOS scores.

All 51 patients in our study returned to their previous employment; however, only 62% returned to high-impact sports. Everhart et al. [21] looked into return to work or sports in a systematic review of 524 patients. They reported that 62% of patients returned to previous work with minimal modification. They also reported that 22-33% returned to high-level sports. Patients having surgical treatment had a higher rate of return to sports than those treated conservatively.

Females were noted to have an inferior outcome in our study. However, we did not observe any differences in terms of demographics, injury patterns, or preoperative scores between males and females, and hence cannot provide a rationale for the difference in results. King et al. [22] have also reported females having inferior outcomes. Larger studies are required to gain more insights into differences associated with gender in MLKI.

We noticed that patients undergoing LCL reconstruction had worse outcomes compared to MCL reconstruction with regard to Tegner scores. This contrasts with the results reported by King et al. [22], who found that patients with medial injuries had worse Lysholm and IKDC scores.

Our study has a few limitations, primarily its small sample size and diversity of injured structures. However, it is very difficult to recruit a homogenous cohort within this population considering associated injuries to the menisci and articular surfaces. We also did not look at radiological outcomes. The main strength of our study is the midterm follow-up and patient-reported outcome measures in the form of subjective scores. We believe that subjective scores are more relevant to clinical practice.

## Conclusions

Based on our findings, good functional results were observed at a median four-year follow-up after delayed ligament reconstruction for MLKI. Female patients and patients with a higher BMI had inferior outcomes, and this should be addressed in preoperative counselling. Studies with longer-term follow-ups are required to determine if the benefits of reconstructive surgery can be maintained in the long run.
